# Mid-term follow-up and outcomes of patients with prosthetic heart valves: a single-centre experience

**DOI:** 10.1186/s44156-022-00001-w

**Published:** 2022-06-06

**Authors:** Sadie Bennett, Polyvios Demetriades, Keely Banks, Jacopo Tafuro, Rosie Oatham, Timothy Griffiths, Cheryl Oxley, Sally Clews, Grant Heatlie, Chun Shing Kwok, Simon Duckett

**Affiliations:** 1 Heart & Lung Centre, Royal Stoke University Hospital, University Hospitals of North Midlands, Newcastle Road, Stoke-on-Trent, UK; 2 Keele University, Stoke-on-Trent, UK

**Keywords:** Echocardiography, Prosthetic heart valves, Patient outcomes

## Abstract

**Background:**

Patients with prosthetic heart valves (PHV) require long-term follow-up, usually within a physiologist led heart valve surveillance clinic. These clinics are well established providing safe and effective patient care. The disruption of the COVID-19 pandemic on services has increased wait times thus we undertook a service evaluation to better understand the patients currently within the service and PHV related complications.

**Methods:**

A clinical service evaluation of the heart valve surveillance clinic was undertaken to assess patient demographics, rates of complications and patient outcomes in patients who had undergone a PHV intervention at our institute between 2010 and 2020.

**Results:**

A total of 294 patients (mean age at time of PHV intervention: 71 ± 12 years, 68.7% male) were included in this service evaluation. Follow-up was 5.9 ± 2.7 years (range: 10 years). 37.1% underwent baseline transthoracic echo (TTE) assessment and 83% underwent annual TTE follow-up. Significant valve related complications were reported in 20 (6.8%) patients. Complications included a change in patient functional status secondary to significant PHV regurgitation (0.3%) or stenosis (0.3%), PHV thrombosis (0.3%) or infective endocarditis (3.7%). Significant valve related complications resulted in ten hospital admission (3.4%), two re-do interventions (0.6%), and four deaths (1.3%).

**Conclusions:**

This service evaluation highlights the large number of patients requiring ongoing surveillance. Only a small proportion of patients develop significant PHV related complications resulting in a low incidence of re-do interventions and deaths.

## Introduction

Valvular heart disease (VHD) is common in the developed world and its prevalence is set to rise with an ever-increasing life expectancy. The gold standard treatment for severe VHD has been prosthetic heart valves (PHV) in the form of surgical valve replacement and/or surgical valve repair [1], more recently percutaneous valve interventions including transcatheter aortic valve implantation has created an opportunity to treat patients that have been previously declined for surgery due to high surgical risk.

PHV interventions required patients to undergo long-term follow-up, regardless of position or interventional technique. However, guidance on the most effective follow-up protocol has changed over recent years from all patients being required to undergo baseline (within 6 weeks of PHV intervention) and subsequent annual transthoracic echocardiogram (TTE) [1], to patients requiring baseline TTE and subsequent “late” follow-up at ≥ 5 years (patients < 50 years) or ≥ 10 years (patients > 50 years) with surgical bio-prosthetic valves (aortic or mitral). Annual TTE assessment for all percutaneous valve interventions and no routine follow-up for mechanical valve replacements was also recommended [2].

Physiologist led heart valve surveillance clinics are well established within clinical practice with evidence suggesting these services to be not only feasible and sustainable [3], but also able to provide safe and cost effective care for patients [4]. At the University Hospitals of North Midlands NHS Trust, a cardiac physiologist led heart valve surveillance clinic was established in 2010. This clinic evaluates patients with native heart valve disease and patients with PVHs. However, numerous reasons including improved treatment options, an ever increasing and aging population and the COVID-19 pandemic has increased the demand for this service resulting in long wait times.

In an attempt to provide an updated overview of current patient cohort with PVH within the service, along with information regarding PHV related complications and patient outcomes, a clinical service evaluation was undertaken. The objective of this evaluation was to determine the current patient numbers with PHV (surgical and percutaneous), adherence to local follow-up protocols and the long term safety of PHV including the development of complications and major adverse cardiovascular events.

## Methods

A retrospective clinical service evaluation using the heart valve surveillance clinic’s patient database was conducted together with extraction of data from a manual search of electronic patient records and a review of all TTEs. Patients were included if they underwent a PHV procedure since 2010 regardless of intervention type (surgical or percutaneous) or valve position. Patients who underwent a PHV procedure prior to 2010 were excluded as the index procedure would have pre-dated the establishment of the heart valve clinic thus adherence to protocols could not be reliably assessed.

Patient age, gender, duration of follow-up, indication for intervention, intervention type (surgical or percutaneous), PVH type and position and reasons for discharge from the valve surveillance clinic were extracted from the valve clinic database. Electronic patient records and TTE studies were reviewed. Patient outcomes that were assessed for included hospital admissions (≥ 1 night for any cause), a reduction in left ventricular ejection fraction (defined as of < 40%, or a reduction by ≥ 10% compared to pre-operative values) and mortality. Significant valve-related complications can be seen in Table 1 and were in accordance with previously published criteria [5]. Due to the expected high number of PVH paravalvular/transvalvular regurgitation, the decision was taken to define PVH regurgitation (paravalvular or transvalvular) as significant if it was associated with a change in a patients functional/symptomatic status (≥ 1 change in New York Heart Association classification). International guidance was used to define significantly elevated transvalvular gradients [6]. In the patients who underwent a baseline TTE assessment and subsequent follow-up, right ventricular systolic pressure (RVSP) and degree of tricuspid regurgitation (TR) was measured at baseline and at the last follow-up undertaken. RVSP was chosen in place of pulmonary artery systolic pressure as it was anticipated that the inferior vena cava could not be reliably assessed for in all patients.

**Table 1:** Prosthetic heart valve related complications*

Prosthetic heart valve related complication	Definition
30-day mortality	Death, of any cause within 30 days of operation regardless of the patient’s geographic location
Valve thrombosis	Thrombosis in the absence of infection attached to or near an operated valve that occludes part of the blood flow path or that interferes with function of the valve
Bleeding event	Any episode of major internal or external bleeding that causes death, hospitalization, or permanent injury (e.g. vision loss) or requires transfusion
Structural dysfunction	Wear and tear, fracture, poppet escape, calcification, leaflet tear, stent creep, and suture line disruption of components (e.g. leaflets, chordae) causing dysfunction of an operated valve
Non-structural dysfunction—stenosis	Abnormality resulting in stenosis or regurgitation of the operated valve (exclusive of thrombus and infection). Examples include: entrapment by pannus, tissue, or suture; paravalvular leak; inappropriate sizing or positioning; residual leak or obstruction from valve implantation / repair, and clinically important hemolytic anaemia
PHV IE	Valvular IE of any infection involving an operated valve. The diagnosis is based on clinical criteria including an appropriate combination of positive blood cultures, clinical signs, and/or histologic confirmation of endocarditis at re-operation or autopsy
PHV IE requiring re-do	Operated valvular IE of any infection involving an operated valve (as per PHV IE) which requires re-do intervention
Re-do for altered PHV function	Re-operation that seeks to repair, alter, or replace a previously operated valve
PHV related mortality	Valve-related mortality is death caused by structural valvular deterioration, non-structural dysfunction, valve thrombosis, embolism, bleeding event, operated valvular IE, or death related to re-operation of an operated valve Deaths caused by heart failure in patients with advanced myocardial disease and satisfactorily functioning cardiac valves are not included

PHV: Prosthetic heart valve, IE: infective endocarditis, re-do: re-do operative

*Taken from Edmunds et al. [5]

As this study was classified as a retrospective single-centre clinical service evaluation, ethical approval from the hospital was not required. The audit was registered with the hospital’s research and development department (Audit Number CA24021). The study was conducted in accordance with the declaration of Helsinki.

## Results

A total of 294 patients who had undergone a PHV procedure since 2010 in our heart valve surveillance clinic database were included in this evaluation. The mean age of the patients at time of the operation was 71 ± 12 years and 68.7% were male. The patients were followed up for an average of 5.9 ± 2.7 years (range 10 years). The indication for surgery and PHV intervention (type and position) are shown in Table 2. The most common indication for surgery was aortic stenosis (67.7%) followed by mitral regurgitation (11.2%) and infective endocarditis (6.5%). 185 (62.9%) patients did not undergo baseline TTE within 6 weeks (before being referred to the valve surveillance clinic). Once enrolled into the valve surveillance clinic, 82.6% had yearly surveillance with clinical and TTE assessment. The remaining 17.4% were followed up less frequently as per the referring consultant’s management plan.

**Table 2:** Indications for surgery, heart valve intervention (type and position) and follow-up data

	*N (%)*
*Indication for surgery*	
Aortic stenosis	199 (67.7)
Mitral regurgitation	33 (11.2)
Infective endocarditis	19 (6.5)
Other	43 (14.6)
*Prosthesis/repair type and position*	
Bioprosthetic—AVR	192 (65.3)
Mechanical—AVR	46 (15.6)
Bioprosthetic—MVR	13 (4.4)
Mechanical—MVR	3 (1.0)
AVR and MVR (bioprosthetic)	4 (1.3)
TAVI	14 (4.7)
MV repair ± annuloplasty ring	26 (8.8)
TVR (with bioprosthetic AVR)	6 (3.1)
TVR (with mechanical MVR)	1(33.3)
TVR (with mitral valve repair ± annuloplasty ring)	3(11.5)
*Follow-up*	
Baseline TTE	109 (37.1)
Annual TTE and clinical review	243 (82.6)

AVR: aortic valve replacement, MVR: mitral valve replacement, MV: mitral valve, TAVI: Transcatheter aortic valve implantation, TVR: tricuspid valve repair, TTE: transthoracic echocardiography

There were 22 patients who were discharged from the valve surveillance clinic. Reasons for discharge included extensive cardiac history and the need for consultant follow-up (N = 11), diagnosis of frailty/dementia which made the patient unsuitable for further intervention (N = 8), and follow-up no longer being required (N = 3). Only 1 patient was lost to follow-up (follow-up care now re-instated).

In the group of patients who underwent a baseline TTE and subsequent yearly TTE follow-up (N = 109, 37.1%), the timeframe between baseline and last follow-up was: 1913 ± 1105 days. Of these patients, baseline TTE demonstrated that 57 patients had no TR, 48 had mild TR and 4 had moderate TR. At the last follow-up, 9 patients developed a mild degree of TR and 10 patients developed moderate TR (an increase from no TR in 1 patient and mild TR in 9 patients) The remaining patients saw no increase in TR severity. There were no patients who were identified as having severe TR either at baseline or follow-up. In the patients who had a measurable RVSP (N = 53), there was no significant difference found between RVSP at baseline and follow-up (26 ± 8.1 mmHg vs 26 ± 9.6 mmHg, p = 0.956).

**Fig. 1:**
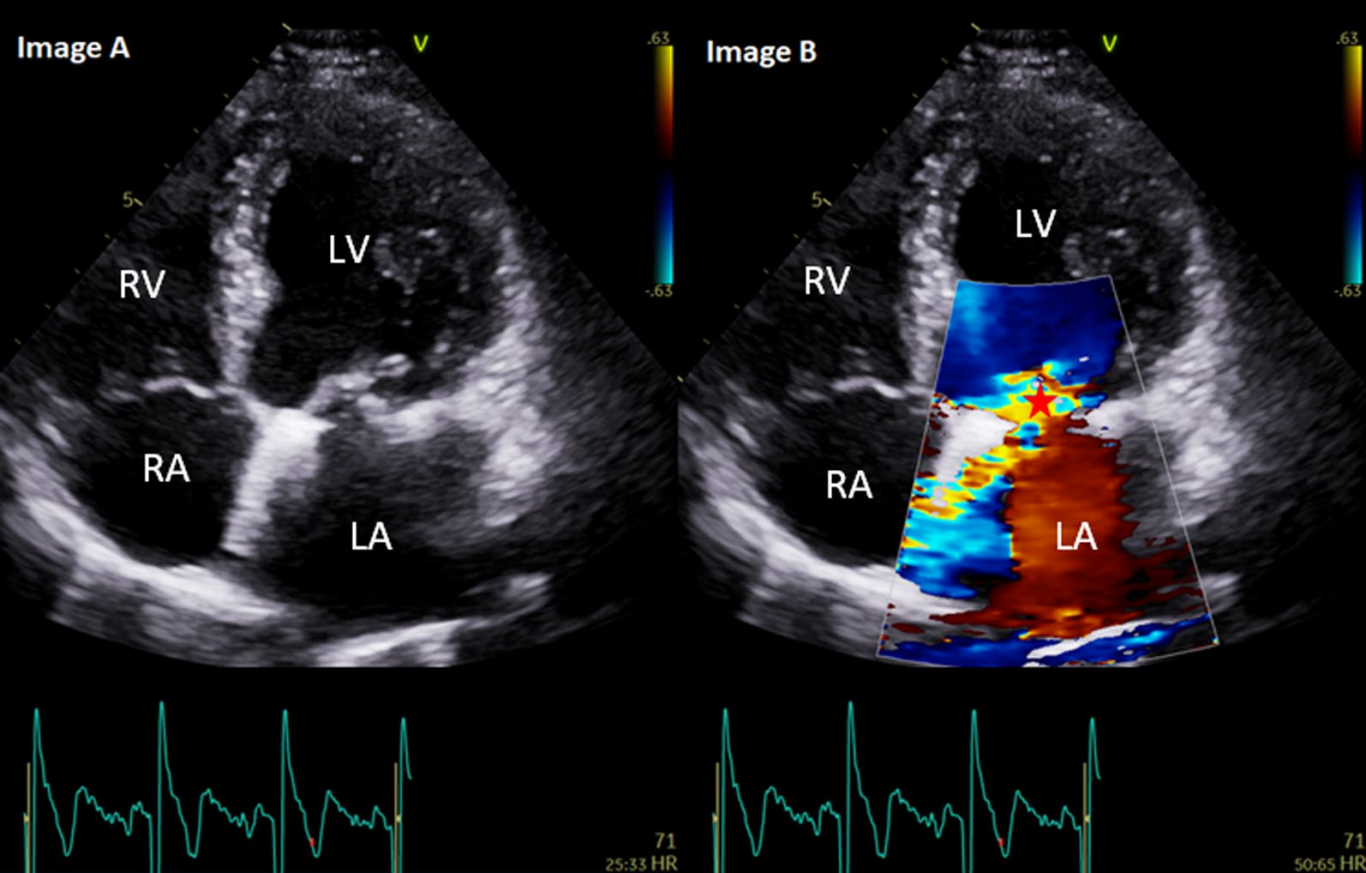
Images** A** and** B**: A 68-year-old male with mitral valve repair + 34 mm annuloplasty ring for severe mitral regurgitation secondary to P2 mitral valve prolapse in 2016. Follow-up in 2019 demonstrated a new finding of moderate, eccentric and anteriorly directed jet of mitral regurgitation (*) secondary to a leaflet co-aptation defect. The left ventricle was mildly dilated by indexed volumes with normal left ventricular systolic function (biplane ejection fraction: 61%). The patient was asymptomatic without any reduction in exercise tolerance. The patient remains on 12 month follow-up. LV: left ventricle, RV: right ventricle, LA: left atrium, RA: right atrium

As shown in Table 3, PHV regurgitation was a common finding post intervention. Mild paravalvular regurgitation was most common in patients with an aortic valve replacement (bio-prosthestic and mechanical) and was identified in 25.5% of all patients with an aortic valve replacement. Re-assuring more severe degrees of PHV regurgitation were rare (see Fig. 1).

**Table 3:** Prosthetic heart valve regurgitation

Valve type	Paravalvular regurgitation N (%)	Transvalvular regurgitation N (%)
AVR—bio and mech. prothesis (N = 238)	Mild: 60 (25.2) Mod: 1 (0.4) Severe: 1 (0.4)	Mild: 5 Mod: 4 Severe: 0 (0)
MVR—bio and mech. prothesis (N = 16)	Mild: 5 (31.2) Mod: 1 (6.2) Severe: 0 (0)	Mild: 0 (0) Mod: 0 (0) Severe: 0 (0)
MV repair ± annuloplasty ring (N = 26)	Mild: 2 (7.6) Mod: 1 (3.8) Severe: 0 (0)	Mild: 7 (26.9) Mod: 3 (11.5) Severe: 0 (0)
TAVI (N = 14)	Mild: 5 (35.7) Mod: 1 (7.1) Severe: 0 (0)	Mild: 0 (0) Mod: 0 (0) Severe: 0 (0)

AVR: aortic valve replacement, MVR: mitral valve replacement, MV: mitral valve, TAVI: Transcatheter aortic valve implantation

### Prosthetic heart valve related complications

**Fig. 2:**
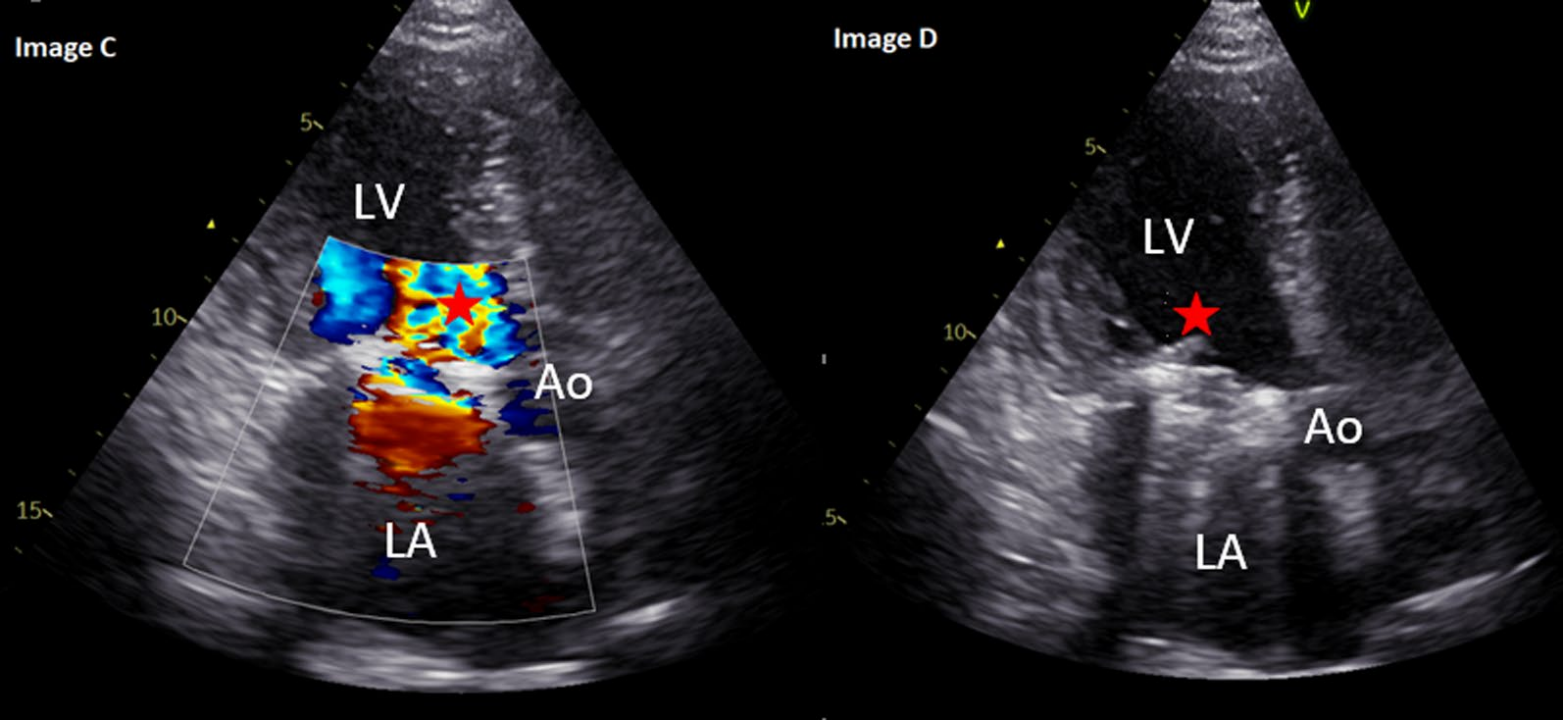
Images** C** and** D**: A 54-year-old female with a mechanical mitral valve replacement in 2012 for severe mitral stenosis secondary to rheumatic fever. In April 2020, the patient presented acutely with fever and night sweats. Blood cultures were positive for Staphylococcus aureus. Transthoracic echocardiography identified a stable in-situ mechanical mitral valve with good occluder mobility. There was turbulent forward flow (* in image C) and significantly elevated transvalvular mean gradient of 15 mmHg (documented as 3.3 mmHg on transthoracic echocardiography 13 months prior). There was a linear mobile mass (* in image D) on the left ventricular size of the mechanical valve replacement which was not visible on previous imaging. There was a high suspicion of infective endocarditis which was confirmed on a subsequent transesophageal echocardiography. The patient was commenced on antibiotic therapy, re-do mitral valve replacement was undertaken 16 weeks later, after which the patient made a good and uneventful recovery. At last follow-up, there was a stable in-situ mechanical mitral valve replacement, mean gradient: 3.4 mmHg, normal left ventricular size and systolic function, biplane ejection fraction: 59%. The patient was asymptomatic without any reduction in exercise tolerance. The patient remains on 12 monthly follow-up. LV: left ventricle, LA: left atrium, Ao: aorta

Significant PVH related complications were identified in 20 (6.8%) of patients (see Table 4). Of these, one patient with a mechanical aortic valve replacement developed PHV thrombosis on day 5 post surgery. This was successfully treated medically with the patient making an uneventful recovery. One patient had PHV re-stenosis, this patient was symptomatic with a change in NYHA classification and required re-do intervention 1339 days post initial bioprosthetic aortic valve replacement. One patient was identified as having severe bioprosthetic (aortic) paravalvular regurgitation with suspected leaflet tear. In this case, the patient was symptomatic with a change in NYHA classification and required re-do intervention 3445 days post initial intervention. Eleven patients developed PHV infective endocarditis (3.7%), two of these patients required re-do valve intervention (see Fig. 2), four patients died, the remaining five patients were treated medically and made an uneventful recovery. The mean time from PHV implantation to diagnosis to infective endocarditis was 1439 ± 690 days. Only one patient had patient prosthetic mismatch and this patient was asymptomatic at the last follow-up appointment and remains on  12 monthly follow-up.

**Table 4:** Prosthetic heart valve related complications

Complication	Valve type and position					
	Biological AVR (N = 192)	Mechanical AVR (N = 46)	Biological MVR (N = 13)	Mechanical MVR (N = 3)	MV repair ± annuloplasty ring (N = 26)	Transcatheter aortic valve implantation (N = 14)
30-day mortality	0 (0)	0 (0)	0 (0)	0 (0)	0 (0)	0 (0)
Valve thrombosis	0 (0)	0 (0)	0 (0)	1 (33.3)	0 (0)	0 (0)
Bleeding event	0 (0)	0 (0)	0 (0)	0 (0)	0 (0)	0 (0)
Structural dysfunction	1 (0.5)	0 (0)	0 (0)	0 (0)	0 (0)	0 (0)
Non-structural dysfunction—stenosis	1 (0.5)	0 (0)	0 (0)	0 (0)	0 (0)	0 (0)
Significant PHV regurgitation with a change in NYHA	1 (0.5)	0 (0)	0 (0)	0 (0)	0 (0)	0 (0)
PHV infective IE	11 (5.7)	0 (0)	0 (0)	0 (0)	0 (0)	0 (0)
PHV IE requiring re-do	1 (0.5)	0 (0)	0 (0)	1 (33.3)	0 (0)	0 (0)
Re-do for altered PHV function	0 (0)	0 (0)	0 (0)	0 (0)	0 (0)	0 (0)
PHV related mortality	4 (2.0)	0 (0)	0 (0)	0 (0)	0 (0)	0 (0)

MV: mitral valve, AVR: aortic valve replacement, MVR: mitral valve replacement, PHV: Prosthetic heart valve, mod: moderate, NYHA: New York Heart Association, IE: Infective endocarditis, re-do: re-operation

### Hospital admissions and left ventricular systolic impairment and mortality

During the follow-up period, 20 patients (6.8%) had a hospital admission of ≥ 1 night stay. The reason for admission in 10 patients was because of infective endocarditis and there were no other PHV related complications resulting in hospitalisation. The other 10 patients were admitted because of non-cardiac conditions which included community acquired pneumonia, exacerbation of chronic obstructive pulmonary disease and other non-valve related infection or sepsis. There were 19 deaths in the evaluated cohort, 4 were a consequence of PHV related infective endocarditis, 11 were due to non-cardiac causes and 9 were due to unknown causes. No patient deaths were recorded within the first 30 days of PHV intervention.

Left ventricular (LV) systolic impairment (≤ 40%) was reported in 19 patients, all of which had normal PHV function at baseline and throughout follow-up. On review of these patients, three patients developed LV systolic impairment during follow-up. Fifteen patients were found to have impaired LV systolic function 6 weeks post PHV intervention with no significant improvement seen during follow-up. Only one patient demonstrated improved LV systolic function at follow-up in comparison to 6 weeks post PVH intervention. Here, LVEF improved from 40% at baseline to 47% at last follow-up (2,876 days).

## Discussion

This service evaluation demonstrated several key findings. There is a large number of patients with PHV who require ongoing long-term care. Most of these patients undergo annual follow-up and few patients are discharged once enrolled. Significant PHV related complications occurs in just over 1 in 20 patients which results in acute hospitalisations for patients. Finally, the results suggests that significant PHV complications occur late on. Overall, these findings suggest that patients with PHV can be safely monitored and managed within a cardiac physiologist led service.

This service evaluation reported a low incidence of primary valve failure with only one patient requiring a re-do intervention. It has been reported that the risk of primary failure of mechanical valves is theoretically extremely low [7]. However, the failure rate of bioprosthetic valves can be as high as 40% at 10 years for those in the mitral position. In the current evaluation, the incidence of infective endocarditis was 0.96% per patient-year and this is comparable to the rate of 0.3–1.2% per patient-year that has been reported previously [8].

This service evaluation also identified that many patients did not have a baseline TTE within the 6-weeks post-operative period. The 6-week timeframe outlined by the British Society of Echocardiography/British Heart Valve Society [2] presents a challenge to implement in clinical practice, as there are long waiting lists for imaging which may result in variations in clinical practice. The lack of adherence to protocols and recommendations within the same institution has been described before. Alaour et al. [9] reported that there was significant heterogeneity in follow-up of patients with PHV with 66% of patients inappropriately discharged following their 6-week baseline assessment and only 19% of patients received the recommended guideline-based follow-up. This highlights the importance of specialist valve clinics which can facilitate a homogeneous and guideline-based approach to managing these patients [10].

Changes in follow-up guidance for patients with PVHs are important to ensure all patients receive the best care. Over the last decade PHV follow-up guidance has changed dramatically moving away from the initially proposed baseline and annual surveillance for all PHV patients [1] to baseline and “late” follow-up being required [2]. The implementation of “late” follow-up for PHV patients may result in a change in their follow-up care, particularly in those patients who have undergone surgical PHV interventions in the last 5 years (aged < 50 years) or 10 years (aged > 50 years). This change in clinical practice will result in a welcome reduction in the number of annual TTEs being required in the short term. However, it will need to be effectively communicated to referring clinicians, general practitioners and patients to ensure they are all informed for the reasons behind this change.

We acknowledge that our study has certain limitations. Firstly, the mean follow-up duration was short which may result in bias when assessing long-term complications. However, the adjustments we implemented in our new protocols relate to the first 5 to 10 years following PHV interventions, at which we show a low incidence of significant PVH related complications. Secondly, our study had a retrospective and single-centre design that only studied a small percentage (10%) of the overall population within the valve surveillance clinic at our centre. However, we have demonstrated a real-world review of a valve surveillance clinic and we believe that our results are reflective of practice across many Cardiology departments in the United Kingdom.

## Conclusion

This service evaluation highlights the large number of patients requiring ongoing surveillance for PVH intervention. Only a small proportion of patients develop significant PHV related complications resulting in a low incidence of re-do intervention and deaths.

## Data Availability

The data that support the findings of this study are available from the corresponding author (SB), upon reasonable request.
